# The relative efficacy of bona fide psychotherapies for post-traumatic stress disorder: a meta-analytical evaluation of randomized controlled trials

**DOI:** 10.1186/s12888-016-0979-2

**Published:** 2016-07-26

**Authors:** Ulrich S. Tran, Bettina Gregor

**Affiliations:** Department of Basic Psychological Research and Research Methods, School of Psychology, University of Vienna, Liebiggasse 5, A-1010 Vienna, Austria

**Keywords:** Post-traumatic stress disorder, Bona fide psychotherapies, Relative efficacy, Meta-analysis

## Abstract

**Background:**

In the treatment of PTSD, meta-analyses suggest comparable efficacy of cognitive behavioural therapies and various trauma focused treatments, but results for other treatments are inconsistent. One meta-analysis found no differences for bona fide therapies, but was critizised for overgeneralization and a biased study sample and relied on an omnibus test of overall effect size heterogeneity that is not widely used.

**Methods:**

We present an updated meta-analysis on bona fide psychotherapies for PTSD, contrasting an improved application of the omnibus test of overall effect size heterogeneity with conventional random-effects meta-analyses of specified treatment types against all others. Twenty-two studies were eligible, reporting 24 head-to-head comparisons in randomized controlled trials of 1694 patients.

**Results:**

Head-to-head comparison between trauma focused and non-trauma focused treatments revealed a small relative advantage for trauma focused treatments at post-treatment (Hedges’ *g* = 0.14) and at two follow-ups (*g* = 0.17, *g* = 0.23) regarding PTSD symptom severity. Controlling and adjusting for influential studies and publication bias, prolonged exposure and exposure therapies (*g* = 0.19) were slightly more efficacious than other therapies regarding PTSD symptom severity at post-treatment; prolonged exposure had also higher recovery rates (*RR* = 1.26). Present-centered therapies were slightly less efficacious regarding symptom severity at post-treatment (*g* = −0.20) and at follow-up (*g* = −0.17), but equally efficacious as available comparison treatments with regards to secondary outcomes. The improved omnibus test confirmed overall effect size heterogeneity.

**Conclusions:**

Trauma focused treatments, prolonged exposure and exposure therapies were slightly more efficacious than other therapies in the treatment of PTSD. However, treatment differences were at most small and far below proposed thresholds of clinically meaningful differences. Previous null findings may have stemmed from not clearly differentiating primary and secondary outcomes, but also from a specific use of the omnibus test of overall effect size heterogeneity that appears to be prone to error. However, more high-quality studies using ITT analyses are still needed to draw firm conclusions. Moreover, the PTSD treatment field may need to move beyond a focus primarily on efficacy so as to address other important issues such as public health issues and the requirements of highly vulnerable populations.

**Electronic supplementary material:**

The online version of this article (doi:10.1186/s12888-016-0979-2) contains supplementary material, which is available to authorized users.

## Background

There is an on-going debate on whether psychotherapies differ in efficacy in the treatment of post-traumatic stress disorder (PTSD). In the broader context of psychotherapy research this is a well-known topic since Rosenzweig [1] introduced the assumption that all psychotherapies are equally effective because of underlying common factors. This topic still remains mostly controversial and unsolved [2], but most notably owing to progress in meta-analytical methodology, some important contributions from an evidence-based point of view could be made for which methodological aspects appeared crucial as well. Whereas early extensive meta-analyses did not make precise distinctions between disorders and outcomes [3, 4], but included different psychotherapies for different disorders, critics warned against overgeneralization, undifferentiated methodology, and confounded results [5–8]. Empirical justification for this critique was provided, for example, in the treatment of anxiety disorders: there, an advantage of cognitive behavioural therapy (CBT) over relaxation treatment pertained in panic disorder but not generalized anxiety disorder, and was evident in primary, symptom-oriented, outcomes, but not in secondary, more general, outcomes [9, 10].

With regards to PTSD, existent meta-analytical reviews suggest that CBT, trauma focused CBT (TFCBT), exposure therapies, and eye movement desensitization reprocessing (EMDR) are equally effective, but results of non-trauma focused therapies like hypnotherapy, psychodynamic therapy or supportive therapy are heterogeneous and inconsistent, meaning either that they are less effective or were yet not sufficiently examined to prove their efficacy [11–21]. Especially the status of supportive therapies appears currently unclear, leaving the question open whether specific techniques really make any difference over common factors [10, 22].

Meta-analytical evidence presented by Benish, Imel, and Wampold [23] fuelled this debate, indicating that any differences in efficacy of psychotherapies for PTSD vanish once the analysis is restricted to head-to-head comparisons, thus controlling for some possible confounders, like differences in diagnostic criteria or length of treatment [8], and includes only ‘bona fide’ psychotherapies, i.e., treatments that are intended to be therapeutic. Bona fide criteria (see the section on Eligibility below) were utilized by this research group also in a number of other meta-analyses [24–26], but were utilized since by some independent other researchers as well [22, 27, 28]. The intention of using bona fide criteria is to restrict the analysis to head-to-head comparisons of active treatments to raise its validity. Including studies with mere ‘intent-to-fail’ control conditions introduces heterogeneity and bias, which may result in spurious differences of treatment efficacy in meta-analysis [23, 29].

The Benish et al. [23] meta-analysis has been critizised for a biased selection of the available evidence and overgeneralization [30]. For example, Cloitre [31] included 44 comparisons from 27 studies, whereas Benish et al. [23] included only 17 comparisons from 15 studies. Specifically, Benish et al. [23] were critizised for including present-centered therapies and therapies whose efficacy appeared debatable, but excluding supportive therapies [30]. Moreover, supportive therapies have been categorized in other meta-analyses either as active treatments [12, 32] or mere control conditions [13, 16]. Application of an operational definition of what constitutes an ‘active treatment’ is therefore needed (e.g., the bona fide criteria), in order to make sense of differing results and conclusions reached by extant reviews and meta-analyses.

However, as of yet, there have been no comprehensive attempts to update and re-evaluate these issues and results empirically. One further meta-analysis [19] examined group treatments for PTSD and found no indication of differences in treatment efficacy; however, this result was based on a sample of ten studies wherein treatments were not necessarily bona fide. Ougrin [15] found no differences between cognitive therapy and exposure therapy, aggregating the evidence of only five studies. Munder et al. [33] examined differences between various trauma focused treatments, but only with regards to their dependency on researcher allegiance. Watts et al. [21] presented a comprehensive meta-analysis on the efficacy of treatments for PTSD, including also pharmacotherapies, but did not strictly rely on head-to-head comparisons and psychotherapies were not specifically bona fide. Tolin [22], focusing specifically on CBT, reported an advantage of CBT over other psychotherapies in the treatment of anxiety and depressive disorders, but not for PTSD. A recently published meta-analysis [14], that was not restricted to head-to-head comparisons, reported for adult survivors of childhood abuse best effects for trauma focused treatments in an individual setting. Therefore, the studies conducted in this area have been mixed in their findings and fairly heterogeneous in their approach.

There is yet one further important aspect of the Benish et al. [23] meta-analysis that did not receive a re-evaluation. Benish et al. [23] used a specific method (see Methods) which was proposed by Wampold et al. [4] in order to circumvent the need for a priori treatment categorizations. Instead of meta-analytically comparing individual classes of treatment against all others, as in other meta-analyses on relative efficacy, Wampold et al. [4] proposed an omnibus test of overall effect size heterogeneity that does not rely on a priori treatment categorizations, but on the random assignment of positive (+) or negative signs (−) to the effect sizes of the head-to-head comparisons. The results of the first meta-analysis which used this approach [4] had a huge impact on the debate on whether psychotherapies really differ in efficacy. However, this test cannot be considered a standard procedure, because it is not commonly used in meta-analytical research on relative efficacy. In fact, it seems that it is used only in meta-analyses of the same researchgroup [4, 23–25, 33]. Therefore, it remains currently unclear whether this method provides results that are consistent with conventional methods. Specifically, low statistical power and the risk of capitalizing on chance may threaten the statistical conclusion validity of this test (see Methods).

The main aim of this meta-analysis was to make a contribution to both the overall field of research on relative treatment efficacy with regards to methodology, and to the specific field of research on the relative efficacy of treatments for PTSD, utilizing advanced meta-analytical methodology. The current study re-addressed the question whether there are no differences in the efficacy of treatments for PTSD, utilizing the bona fide criteria for an operational definition of ‘active treatments’, updating the available evidence, and evaluating the study selection and methodology used by Benish et al. [23] (see Methods section). Specifically, we improved the application of the proposed statistical method and contrasted its results with conventional meta-analysis of direct comparisons, and also explored whether trauma focused treatments were in direct head-to-head comparisons more efficacious than treatments that were non-trauma focused. Moreover, we also investigated effects of patient and treatment variables on relative efficacy and controlled for study quality.

## Methods

The methodology of this study adhered to PRISMA guidelines.

### Eligibility

Eligibility criteria for studies to be included in this meta-analysis were (see [23]): (1) a randomized and controlled study design (RCT), investigating the relative efficacy of (2) at least two bona fide psychotherapies; (3) therapies needed to be conducted in two or more sessions; (4) participants were adults that were (5) diagnosed with PTSD according to the valid edition of the DSM at the time of the respective study (DSM-III or DSM-IV); (6) PTSD symptom severity was assessed with self-report or clinician rating. Studies that provided insufficient information to compute effect sizes were excluded as were component, dismantling, and parameter studies. Moreover, we also excluded studies that contained mere additional analyses on previously published data, ensuring that primary data entered analysis only once.

Target group, treatment format and study quality did not serve as eligibility criteria in the present study. In order to examine the possible effect of various patient and treatment characteristics as well as the influence of study quality, moderator analyses were conducted (see the section on Additional Analyses, below). Taylor and Harvey [32] examined the effects of psychotherapy specifically for people who have been sexually assaulted and Ehring et al. [14] for adult survivors of childhood abuse. Sloan et al. [19] and Bisson et al. [12] examined, amongst others, group therapies. However, other meta-analyses did not limit themselves to specific target groups or treatment formats [13, 16–18, 20, 34].

To qualify as an ‘active treatment’, the bona fide definition [23, 29] required that treatments had to be delivered by a trained therapist who adapted the treatment to patients on the basis of a therapeutic relationship (i.e., no delivery of a non-modifiable standard protocol, e.g., progressive muscle relaxation); treatments also needed to be conducted personally and face-to-face (i.e., no online treatments or treatments conducted with, e.g., audio material). Moreover, at least two of the following four criteria had to be fulfilled with regards to their descriptions in the studies: (a) a citation to an established school or approach to psychotherapy; (b) a description of the therapy that contained a reference to a psychological process (e.g., operant conditioning); (c) a reference to a treatment manual that was used to guide the delivery of the treatment; (d) the identification of active ingredients of the treatment and citations for these ingredients.

### Information sources and search

**Fig. 1:**
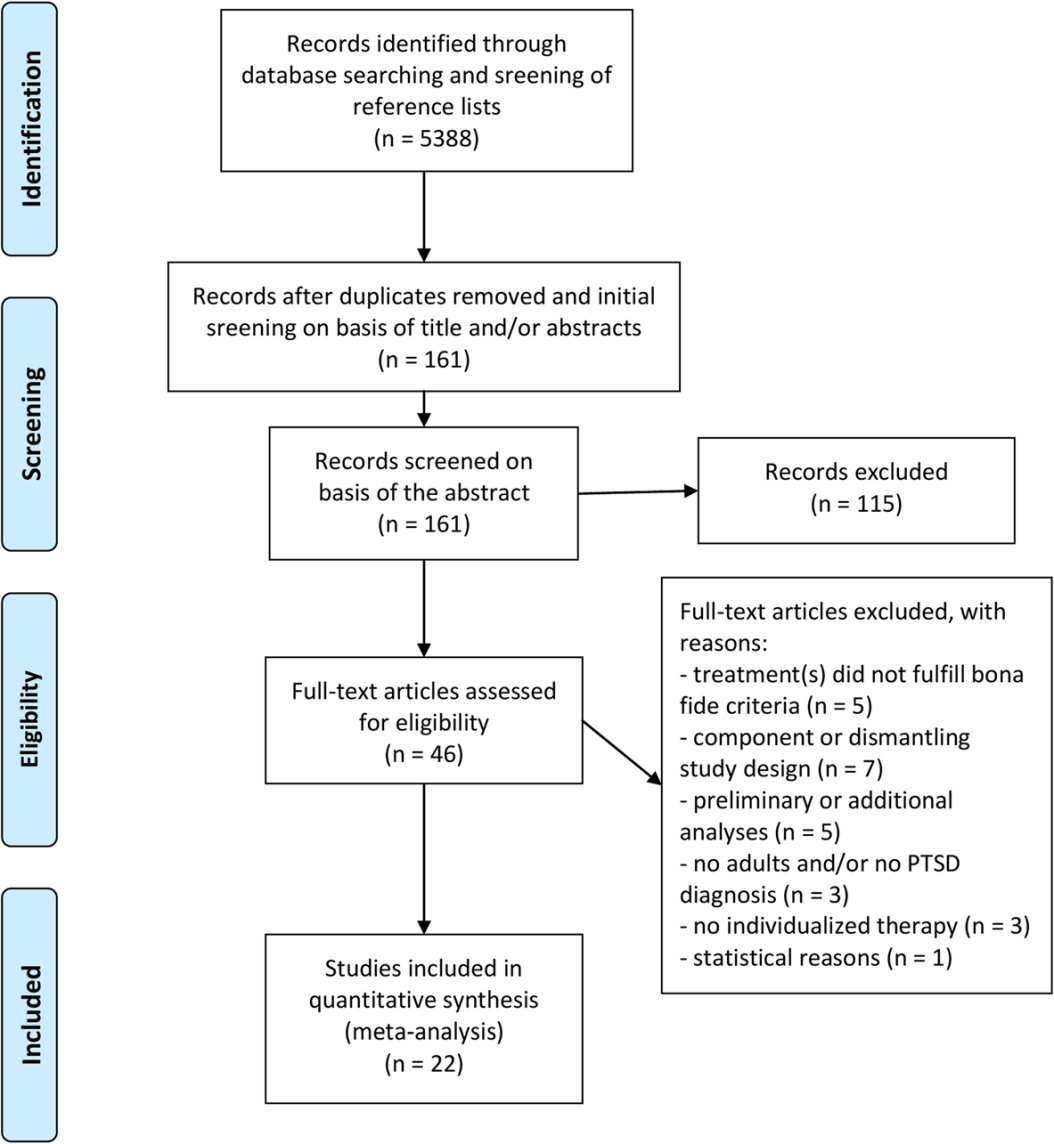
Flow of studies through the selection process

Primary studies were identified in two ways. We obtained the 15 studies investigated by Benish et al. [23] and performed a literature search in the databases MEDLINE, Cinahl Health, Psychology and Behavioral Sciences Collection, PsycINFO, PsycArticles, PubMed, Social Sciences Fulltext, and Web of Knowledge, using the keywords *PTSD*, *posttraumatic*, *post-traumatic*, *post traumatic* in combination with *psychotherapy* and *treatment*, and also in combination with *relative efficacy*, *outcome study* and *comparative efficacy*. We refined the search results by restricting it to ‘adults’, ‘humans’ and ‘randomized controlled trials’ in some databases (see Fig. 1). In addition, we searched reference sections of previous meta-analyses of PTSD treatment, controlled studies of psychotherapy outcomes for PTSD, and literature reviews of PTSD treatment [31, 35, 36].

Moreover, we meticulously re-assessed the bona fide status of all treatments included in previous meta-analyses and of the treatments of the 27 studies included in the review of Cloitre [31]. This review was specifically mentioned by Ehlers et al. [30] in their criticism with regards to a biased study sample of Benish et al. [23].

### Study selection and data collection

Prior to study selection, descriptions of 112 treatments classified as bona fide in previous meta-analyses on PTSD [23], alcohol abuse [24], and depression [26] as well as descriptions of treatments not classified as bona fide in these study samples were screened to ensure that we applied the bona fide criteria correctly. Moreover, we closely adhered to the descriptions and examples provided by [29].

The second author extracted the data and coded the studies. Cases where coding appeared unclear were resolved by discussion and consensus between both authors. Moreover, coding of included studies was compared afterwards with coding provided in other meta-analyses for the same studies, where available [13, 16, 17, 32, 35]; in these cases, no differences in coding were observed. Wherever possible, intention-to-treat (ITT) analyses were used in meta-analytical computations.

### Data items

Coded and extracted data items pertained to (a) study characteristics: publication year, type of publication, country, type of analysis (ITT or completer only); (b) patient characteristics: total number, number per treatment arm, percentage of women, mean age, type of trauma, mean time since trauma, PTSD symptom severity prior to treatment, percentage of comorbid disorders (depression, anxiety, substance use disorder), site of patient recruitment (community, clinical, community and clinical mixed, other), use of psychotropic drugs; (c) treatment characteristics: type of treatment, treatment format (individual vs. group), treatment length, total number of sessions, session length; and (d) all relevant data with regards to the primary and secondary outcomes, clinically significant change, and dropout (see the section on Summary Measures, below).

### Study quality

Risk of bias was evaluated on the basis of the seven criteria of the *Practice Guidelines from the International Society for Traumatic Stress Studies* [37], complemented by standards of high-quality studies proposed by Cuijpers et al. [38]. Study quality was evaluated with respect to whether (1) randomization was conducted adequately and independently from researchers and therapists (by using, e.g., computer software); (2) assessment was blinded; (3) studies reported reasons for dropout or analyses on differences between dropouts and completers; (4) therapists were specifically trained in the provided treatment; (5) adherence to treatment manuals or protocols was ensured with adequate measures or checked empirically; (6) ITT-analyses were conducted and adequately reported; (7) a treatment manual or protocol existed. Items were coded 0 = *no*, 1 = *yes*, and summed, resulting in an omnibus measure of study quality that ranged from 0 to 7. Additionally, we also investigated criteria (1), (2), and (3) separately. These criteria agree closely with the Jadad et al. [39] criteria^1^ that are widely-used in medical research and that were already also used in psychotherapy research [16, 22]; with regards to blinding, we considered only blinded assessment; double-blind trials, as proposed by Jadad et al. [39] are adequate in medical research, but are generally not feasible in psychotherapy research.

### Summary measures

First, between-group differences in primary and secondary outcomes (see below) were meta-analysed at post-treatment and, where available, at follow-up. Benish et al. [23] restricted their analyses to post-treatment differences. All direct measures of PTSD symptom severity (e.g., Clinician Administered PTSD Scale [CAPS], Impact of Events Scale [IES], PTSD Symptom Scale Self-Report [PSS-SR]) were considered primary outcomes. This was in contrast to Benish et al. [23] who considered measures of anxiety (e.g., State-Trait Anxiety Inventory [STAI]) primary outcomes as well. Anxiety is one of the key symptoms of PTSD and anxiety disorders are among the most frequent comorbid disorders of PTSD [37]. However, measures of anxiety do not address the full range of PTSD symptoms. Hence, they were not considered primary outcomes in the present study. On the other hand, Benish et al. [23] computed a second effect size, aggregating all post-treatment outcome measures that were reported in individual studies. As these included primary outcomes as well, there was a substantial overlap of this second effect measure with the primary outcome measure. Instead of following such an approach, we considered all measures of symptom severity of comorbid disorders (i.e., anxiety, depression, substance use disorder), trauma-related symptoms (e.g., trauma-related guilt), general symptom distress, social functioning, and quality of life as secondary outcomes, thus having a true second measure for a more differentiated analysis, see [6, 9, 10]. Following [12], we also distinguished primary outcomes with regards to self-reports and clinician ratings. While the meta-analysis of Van Etten and Taylor [20] did not find any differences in efficacy with regards to assessment, others did [32, 40]. A complete list of primary and secondary outcome measures is displayed in Appendix (Tables 5 and 6).

Second, additional outcomes pertained to treatment differences in percentages of clinically significant changes and dropouts at post-treatment and, where available, at follow-up. Where available, recovery rates were used to assess clinically significant change. Otherwise, rates of participants improved were utilized. As in the analysis of primary and secondary outcomes, data of ITT analyses were used, where available. Dropout rates were calculated either on patients dropping out of treatment or on the basis of ITT participant numbers (i.e., after randomization).

### Categorization of treatments

Based on descriptions and references given in the respective studies, treatments were categorized into specific types that were in line with previous meta-analyses [13, 16, 17] and a review [31]. Treatment categorization in these studies followed a bottom-up strategy, sorting and clustering treatments according to their provided descriptions consistently into groups. The present meta-analysis also adhered to this bottom-up treatment categorization scheme, adhering as closely as possible to treatment descriptions in studies for the categorization of treatments. Similar to previous meta-analyses of PTSD comparing several treatment categories, categories resulting from this process were sometimes narrower and sometimes broader, depending on whether therapies followed a specified treatment manual (e.g., eye movement desensitization and reprocessing [EMDR] [41]), or were combined because of a similar theoretical approach (e.g., exposure therapies). In order to maximize statistical power, we were careful not to narrow categories down too much, as this would have yielded a large number of categories and only few studies per category. To compensate for this broadening of categories, we conducted also meta-analyses for distinct subgroups (see below) and controlled for outliers.

Types of treatment included in the analyses were: Exposure therapies (EX), prolonged exposure (PE [42]), and both combined (EXPE), EMDR [41], cognitive behavioural therapies (CBT), and present-centered therapies (PCT). PCT included bona fide psychotherapies that were mostly linked to elements of Roger’s client-centered psychotherapy [43] and encompassed supportive, present-centered, problemsolving, and psychoeducative elements. Treatments coded as CBT included some therapies that utilized, but did not primarily rely on, elements of other therapies (e.g., exposure [44, 45]), and treatments such as *Seeking Safety* [46] or *Image Rehearsal* [47].

All treatments were coded whether they were trauma focused (TF) or not (NTF). TF treatments were defined as focusing on the memory for the traumatic event and/or on trauma-related appraisals [12]. Najavits and Hien [48] recommended to name non-trauma focused therapies for what they are providing and not just to qualify them for what they are not providing. Nevertheless, we adhered to TF/NTF distinction as it is frequently used in this field of research. The TF/NTF distinction served to strictly compare two categories on the same level of abstraction and to address the question of whether trauma focused treatments were more efficacious than non-trauma focused ones. Again, treatment descriptions in studies were utilized as closely as possible to code whether treatments were trauma focused or not. Within the CBT category we also examined the efficacy specifically of trauma focused treatments that included elements of exposure (TFCBT + EX).

### Synthesis of results

For primary and secondary outcomes, Hedges’ *g* was used as effect size measure, similar to previous meta-analyses [16–18, 32], but in contrast to Benish et al. [23] who used Cohen’s *d*. Hedges’ *g* is widely recommended in the meta-analytical literature to control for the overestimation of effects with Cohen’s *d* in small samples and its use is also recommended in the Practice Guidelines from the International Society for Traumatic Stress Studies [37]. In cases where studies reported more than one outcome (primary or secondary), effect sizes were obtained for each outcome and then averaged within outcomes; dependency between outcomes was accounted for by assuming a correlation of .50 in the computation of the variance of the averaged effect size as in Benish et al. [23]. In cases where both full-scale and subscale scores of a measure were reported, full-scale scores were used. In cases where only subscale, but not full-scale, scores were reported, published coefficients on the intercorrelation of subscales was used instead of assuming a uniform correlation of .50 in computing the averaged effect size variance (e.g., for the IES–revised form, coefficients were taken from [49], p. 1491). For additional outcomes, percentages of clinically significant changes and dropouts, risk ratios (*RR*) were computed and used in analysis.

For all analyses, random-effects models were utilized, assuming that treatment differences were not represented by a single population effect. With regards to analyses themselves, two independent approaches were utilized: (1) conventional meta-analysis of direct comparisons, contrasting specified types of treatment against all other available treatments, restricted to types with five or more comparisons, see [38], contrasting also trauma focused versus non-trauma focused treatments, and contrasting further all exposure-based treatments (EXPE) against all other treatments. The conventional analyses comprised comparisons regarding primary and secondary outcomes, clinical significance, and dropout as well as analyses of publication bias, sensitivity analyses, and moderator analyses; (2) the Wampold homogeneity test (WHT) with regards to primary and secondary outcomes at post-treatment [4, 50].

For conventional meta-analyses, the software metafor [51] in R was used. Heterogeneity was tested with the *Q* test and assessed with the *I*^2^ statistic that describes the ratio of true heterogeneity to the total observed dispersion with a range of 0 % to 100 %, reporting also 95 % confidence intervals. Heterogeneity was assumed small for *I*^2^ = 25 %, moderate for *I*^2^ = 50 %, and high for *I*^2^ = 75 % [52].

With regards to the interpretation of standardized mean differences, i.e., Hedge’s *g*, we adhered to benchmarks of Cohen [53] interpreting *g* = 0.20 as small, .50 as medium, and 0.80 as large effects. For the interpretation of whether effects are likely clinically meaningful, we adhered to Ferguson [54] and the NICE Guideline Development Group [12] who proposed thresholds of *g* = .41 and *g* = .50, respectively. With regards to the interpretation of risk ratios pertaining to recovery/improvement rates and dropout, we adhered to thresholds devised by the NICE Guideline Development Group [12], regarding risk ratios ≤ 0.80 or ≥ 1.25 as clinically meaningful. Where 95 % confidence intervals of risk ratios included these thresholds, results were interpreted as ‘limited evidence’ of differential efficacy. Significance was set to *p* < .05.

The omnibus hypothesis, whether there were any differences in treatment efficacy overall, regardless of treatment categorization, was investigated with the WHT. The WHT requires in a first step the random assignment of a positive (+) or negative sign (−), in equal proportion [4], to the effect sizes of the head-to-head comparisons. The aggregated effect of the randomly aligned treatment differences will approach zero if *k*, the number of comparisons, is large, but this is not of direct interest in the WHT. In the second, crucial, step, the homogeneity of the obtained effect size distribution is investigated via the *Q* test. If the null hypothesis of no overall treatment differences is not true, then there should be a disproportionate number of observations in the tails of the distribution [4]. Therefore, if homogeneity has to be rejected, the null hypothesis of no overall treatment differences must also be rejected. This null hypothesis has been tested with the WHT in a number of previous analyses [4, 23–25] and has never been rejected.

However, two aspects may compromise the validity of the WHT: (1) the *Q* test is known for its low test power when *k* is small [52]; in the meta-analysis of Benish et al. [23] *k* was 17, heterogeneity may thus have been overlooked in previous research; (2) the WHT relies on only one run of random sign assignment. As stated above, random assignment need not result in an aggregated effect size of null; the probability of this event approaches unity only for *k* → ∞. Calculation of the *Q* statistic depends on the aggregated effect size. Random fluctuations of the aggregated effect size thus affect also the *Q* test of the WHT. Performing the WHT only once for a given dataset may therefore capitalize on random fluctuations whose possible effect is greater for small *k*. In conclusion, the *Q* test of the WHT cannot be considered an exact test, and *Q* values, as well as *I*^2^ values derived from this *Q* statistic, cannot be considered exact statistics of effect size heterogeneity.

In the present study, we sought to overcome this problem by applying the WHT repeatedly on 30 independent runs of random sign assignments, which can be thought of as a Monte Carlo permutation test. Monte Carlo permutation tests are akin and asymptotically equivalent to (exact) permutation tests (i.e., Fisher’s exact test) which obtain the distribution of a test statistic under the null hypothesis by calculating all possible values of this statistic by rearranging (i.e., permuting) the observed data in all possible ways. *P* values are obtained by computing the proportion of test statistic values that are greater than the test statistic of the observed data. Monte Carlo permutation tests sample only a number of permutations and do not perform all possible permutations. In the present case and with regards to the WHT, no clearly defined test statistic could be computed for the observed data: as explained above, the *Q* statistic of the WHT is itself not exact, but intrinsically fuzzy and subject to random fluctuations. However, the principles of Monte Carlo permutation tests could be applied to sample the distribution of *Q* values of the WHT and to obtain the number of significant *Q* tests for a given data set. Instead of relying on the *p* value of a single WHT, we counted the number of *p* values that were less than .05 among 30 replications of the WHT. Assuming the null hypothesis of no overall treatment differences is true, we expected 1.5 significant results (5 % of 30) with a nominal α of .05. To determine whether the null hypothesis of the WHT had to be rejected, the obtained number of significant results among the 30 replications was compared to this expected value, using a binomial test. This test had a power of more than 95 % to reject the null hypothesis of the WHT of no overall treatment differences, if the true number of significant *Q* values was at least 7.5 (i.e., 25 % of *Q* tests were significant).

### Publication bias

Publication bias in meta-analysis has to be investigated in order to check whether studies in the analysis are representative of the overall population of completed studies. A systematic bias could occur either because of one-sided search-strategies or because small studies or studies without significant results were not published on a regular basis. Publication bias was investigated with the trim-and-fill procedure [55] with regards to all meta-analyses of direct comparisons, using metafor in R.

### Additional analyses

#### Dependencies in the data

One of the studies (see Results) contained three comparisons. In order to test and correct for this dependence, all meta-analyses of direct comparisons involving these three comparisons were repeated, keeping only the head-to-head comparison with the largest effect. Three other studies (see Results) compared treatments of the same type. Meta-analyses of direct comparisons involving these studies were repeated as well, excluding these studies.

#### Sensitivity analyses

Furthermore, overall stability of meta-analyses of direct comparisons was examined with sensitivity analysis using the leave-one-out method, investigating whether there were influential outliers in the data. All of these analyses were used to investigate the robustness of obtained results and to adjust for possible outliers that may otherwise bias and distort results.

#### Moderator analyses

Moderator analyses were carried out for meta-analyses of direct comparisons to explain unobserved heterogeneity, using mixed-effects models in cases where more than ten comparisons were available [38], testing for effects of study quality [12, 16], type of analysis (ITT vs. completer only) [12], participant sex (male vs. female vs. mixed) and proportion of women [12, 19, 21], type of trauma [19, 21], time since trauma [16], PTSD symptom severity prior to treatment [56] (severe vs. extreme), and treatment length and total number of sessions [16, 32].

## Results

### Study selection and study characteristics

In addition to the 15 studies included in Benish et al. [23], we retrieved seven further studies that fulfilled the inclusion criteria, resulting in a total sample of 22 studies [42, 44–47, 57–73] (as the re-assessment of the study selection is an important topic of interest, see Additional file 1 for full references of the excluded studies). In sum, 161 abstracts were thoroughly screened and 46 studies assessed in full-text for eligibility, see Fig. 1.

Studies were excluded because they (reference numbers pertain to the reference list in Additional file 1: (a) compared treatments that were not bona fide [1–5]^2^; (b) were component or dismantling studies [6–12]^3^; (c) reported preliminary analyses of studies that were not included in the present meta-analysis themselves [13]; (d) reported additional analyses on data of studies already included in the present meta-analysis [14–17]; (e) did not examine adults and/or did not ascertain a PTSD diagnosis according to DSM-III or DSM-IV [18–20]; (f) included treatments that were delivered in a standard protocol that could not be adapted to individual patients [21–23]; (g) examined only two participants in one treatment arm [24]. This study [24] was excluded on statistical grounds as estimates of population variance, on which Hedges’ *g* depend, are undefined when *n* < 3.

From the studies included in the review of Cloitre [31], only 3 studies remained which fully met the inclusion criteria and therefore were also included in the present meta-analysis [46, 65, 71]. Deviating from Benish et al. [23], the Schnurr et al. [71] study was included. This study investigated a PCT about which Wampold et al. [29] argued it was not bona fide because it bore little resemblance to PCT commonly applied in practice. However, according to the information provided in the study itself, the treatment fulfilled enough criteria (b, c, and d; see the section on Eligibility, above) to be considered bona fide. Moreover, Benish et al. [23] included another study of the same authors [70] that contained basically the same treatment, only delivered in a group format. On these premises, the Schnurr et al. [71] study was therefore included.

Basic characteristics of the 22 included studies, quality ratings, and individual effect sizes of primary and secondary outcomes are displayed in Table 1. Overall, 1694 patients participated in the studies. Studies were published between 1989 and 2010 and 18 (82 %) were conducted in English-speaking countries, 11 of which in the United States. Patients’ mean age ranged from 21.5 to 59.4 years, seven studies contained only women (total *n* = 664), three studies only men (total *n* = 460), in the remaining 12 studies (total *n* = 570) on average 56.0 % of patients were female. Twelve studies included patients with a specific trauma history or otherwise specific patient characteristics (total *n* = 1175): Childhood sexual abuse and adult rape victims (five studies, total *n* = 305), war veterans (four studies, total *n* = 744), fugitives (two studies, *n* = 51), comorbid substance use disorder (one study, *n* = 75). Fourteen studies provided information on time since trauma, which ranged from 1.2 to 22.9 years, with a mean of 7.1 years. With regards to PTSD symptom severity prior to treatment, eleven studies included severely traumatized patients (total *n* = 902; CAPS: 60–79, IES > 43), five studies included extremely traumatized patients (total *n* = 556; CAPS > 79). The remaining six studies did not report on severity or used instruments that provide no cut-offs with regards to severity (i.e., PDS, PSS-I, PSS-SR, PCL).

**Table 1:** Characteristics of included studies

Study	*n* per study arm (dropout)	Compared treatments (plus categorization)	Study quality & Jadad score	Primary outcome (at post-treatment)	Secondary outcome (at post-treatment)
Brom et al. [57]	31(4)/29(4)/29(4)	Trauma desensitization (EX; TF) vs. brief psychodynamic therapy^a^ vs. hypnotherapy^b^ (EX; TF)	0/0/+/+/0/0/0: 1	1 vs. 2: −0.26 [−0.80, 0.29]	1 vs. 2: 0.23 [−0.19, 0.65]
				1 vs. 3: −0.26 [−0.81, 0.28]	1 vs. 3: 0.02 [−0.40, 0.43]
				2 vs. 3: −0.05 [−0.60, 0.51]	2 vs. 3: −0.22 [−0.64, 0.21]
Bryant et al. [58]	31(8)/28(6)	Imaginal EX (TF) vs. in vivo EX (TF)	+/+/+/+/+/+/+: 4	0.05 [−0.37, 0.47]	0.16 [−0.25, 0.58]
Cook et al. [47]^c^	61(22)/63(12)	Imagery Rehearsal (CBT; TFCBT + EX; TF) vs. Sleep & Nightmare Management (CBT)	+/+/+/+/+/+/+: 4	−0.10 [−0.41, 0.20]	−0.09 [−0.36, 0.18]
Cottraux et al. [59]^c^	31(4)/29(14)	CBT (TFCBT + EX; TF) vs. Rogerian supportive therapy (PCT)	+/+/+/+/0/0/+: 4	−0.05 [−0.68, 0.58]	−0.16 [−0.63, 0.30]
Devilly & Spence [60]	15(3)/17(6)	Cognitive-behavior trauma treatment protocol (CBT; TFCBT + EX; TF) vs. EMDR (TF)	0/0/+/+/+/0/0: 1	−0.58 [−1.24, 0.09]	−0.29 [−0.96, 0.39]
Foa et al. [42]^c^	17(3)/14(4)	Stress inoculation training (CBT) vs. PE (TF)	0/+/+/+/+/0/+: 2	−0.54 [−1.37, 0.29]	−0.22 [−0.89, 0.44]
Foa et al. [61]^c^	26(7)/25(2)	Stress inoculation training (CBT) vs. PE (TF)	0/+/+/+/+/0/+: 2	0.14 [−0.46, 0.75]	0.62 [−0.08, 1.16]
Hien et al. [46]^c^	41(16)/34(10)	Seeking Safety (CBT) vs. relapse prevention (CBT)	0/0/0/+/+/+/+: 0	0.32 [−0.04, 0.69]	0.18 [−0.27, 0.64]
Ironson et al. [62]	10(0)/12(6)	EMDR (TF) vs. PE (TF)	0/0/+/0/+/0/+: 1	−0.62 [−1.55, 0.31]	−1.29 [−2.30, −0.28]
Lee et al. [63]	13(1)/13(1)	Stress inoculation training + PE (CBT; TFCBT + EX; TF) vs. EMDR (TF)	0/0/0/+/+/0/+: 0	0.53 [−0.14, 1.19]	0.52 [−0.30, 1.33]
Marks et al. [64]	19(1)/23(3)	Cognitive restructuring (CBT; TF) vs. PE (TF)	0/+/+/0/+/0/+: 2	0.07 [−0.55, 0.70]	0.19 [−0.34, 0.72]
McDonagh et al. [44]^c^	29(12)/22(2)	CBT (TFCBT + EX; TF) vs. problem solving therapy (PCT)	0/+/+/+/+/+/+: 2	0.22 [−0.33, 0.78]	0.22 [−0.21, 0.64]
Neuner et al. [65]^c^	17(2)/14(1)	Narrative exposure therapy (CBT; TFCBT + EX; TF) vs. supportive counselling (PCT)	+/+/+/+/+/0/0: 4	−0.06 [−0.80, 0.68]	−0.23 [−0.98, 0.52]
Paunovic & Öst [66]	10(3)/10(1)	CBT (TFCBT + EX; TF) vs. EX (TF)	0/0/+/0/0/0/0: 1	−0.20 [−0.94, 0.54]	−0.29 [−1.05, 0.47]
Power et al. [45]	37(16)/39(12)	Cognitive restructuring + EX (CBT; TFCBT + EX; TF) vs. EMDR (TF)	+/+/+/+/+/0/+: 4	0.55 [0.07, 1.02]	0.56 [0.10, 1.02]
Ready et al. [67]^c^	6(1)/5(1)	Virtual reality EX (TF) vs. PCT	0/+/+/0/0/0/+: 2	−0.51 [−1.86, 0.84]	−1.62 [−3.26, 0.02]
Resick et al. [68]	62(11)/62(12)	Cognitive processing (CBT; TFCBT + EX; TF) vs. PE (TF)	0/0/+/+/+/+/+: 1	−0.27 [−0.57, 0.04]	−0.46 [−0.74, −0.18]
Rothbaum et al. [69]	25(5)/23(3)	EMDR (TF) vs. PE (TF)	0/+/0/0/+/0/+: 1	0.34 [−0.17, 0.85]	0.50 [0.00, 1.00]
Schnurr et al. [70]^c^	162(44)/163(28)	Trauma-focused group therapy (CBT; TFCBT + EX; TF) vs. PCT group therapy	+/+/+/0/+/+/+: 4	−0.12 [−0.31, 0.07]	0.00 [−0.17, 0.17]
Schnurr et al. [71]^c^	143(30)/141(53)	PCT vs. PE (TF)	+/+/+/+/+/+/+: 4	0.31 [0.11, 0.51]	0.14 [−0.05, 0.33]
Tarrier et al. [72]	37(4)/35(6)	Cognitive therapy (CBT; TF) vs. imaginal EX (TF)	+/+/0/0/+/0/+: 3	0.17 [−0.22, 0.57]	0.07 [−0.36, 0.50]
Taylor et al. [73]	19(4)/22(7)	EMDR (TF) vs. EX (TF)	0/+/+/+/+/0/+: 2	0.32 [−0.30, 0.94]	0.17 [−0.40, 0.74]

*Note*. ^a^Not categorized with regards to type of treatment; ^b^categorized according to description given in study; ^c^used for comparison of TF versus NTF therapies. Figures of study quality correspond to ratings (+: present; 0: absent) whether (1) randomization was adequate, (2) assessment was blinded, (3) reasons for dropout or analyses on differences between dropouts and completers were reported, (4) therapists were trained in the provided treatment, (5) adherence to treatment manuals or protocols was ensured with adequate measures or checked empirically, (6) ITT-analyses were conducted and adequately reported, (7) a treatment manual or protocol existed (in this order); Jadad scores (min = 0, max = 4), based on these ratings, are reported after the colon. Outcomes correspond to estimates of Hedges’ *g* along with 95 % confidence intervals; *g* > 0 signifies that effects at post-treatment were larger for the first of the two compared treatments, *g* < 0 signifies that effects were smaller

Details regarding the categorization of treatments in the present meta-analysis and in previous meta-analyses are reported in Appendix (Table 7). The psychodynamic therapy investigated in Brom et al. [57] was neither categorized with regards to type of treatment, as it did not fit with any type, nor included in direct head-to-heads comparisons of TF and NTF treatments as it remained unclear whether or not it was trauma focused [29]; it served only as an available comparator in meta-analyses of direct comparisons of specific treatment types. The hypnotherapy investigated in Brom et al. [57] was categorized as EX, as the description in the study explicitly stated that “The emphasis of the hypnotherapists in our study was on behavioral therapy. The goal was to bring the patient in contact with the reality of the traumatic event and to bring about a decrease in the conditioned responses triggered by the event” (p. 607) and that hypnosis was used to facilitate this goal (meta-analyses [12, 13] coded this treatment as ‘other’ treatment). The Hien et al. [46] study investigated the efficacy of Seeking Safety, a manualized CBT that addresses both PTSD and substance use disorder, comparing it with another CBT (relapse prevention) that addresses only substance use disorder. Both treatments were coded as CBT, but in the CBT related meta-analyses only Seeking Safety was pooled with the other CBT treatments for PTSD (and contrasted with relapse prevention), as relapse prevention did not directly address the PTSD symptomatology.

Twenty-four head-to-head comparisons were reported in the 22 studies; one study compared three treatments. There were 15 comparisons involving CBT (of these, 10 were TFCBT + EX), seven involving EX, seven involving PE, six involving EMDR, and six involving PCT. Therapies were conducted in 12 sessions that lasted 90 min on average. Only four (8.9 %) treatments were carried out in a group format. Follow-up assessments were reported in all (100 %) studies with regards to at least one of the defined outcomes; time of follow-up ranged from 1 to 15 months after end of treatment, 4.7 months on average. Nine (40.9 %) studies reported a second follow-up, ranging 6 to 24 months after end of treatment, 10.0 months on average.

### Study quality

Ratings of study quality are presented in Table 1. Most studies reported reasons for dropout or reported analyses of differences between completers and dropouts, ensured adherence to treatment manuals or protocols, and provided a treatment manual or protocol (18 [81.8 %] studies each). Fifteen (68.2 %) studies each reported blinded assessment and whether therapists were specifically trained in the provided treatment. Only eight (36.4 %) studies reported adequate randomization, whereas only seven (31.8 %) intention-to-treat analyses. Jadad ratings ranged between 0 and 4 points, with 2.2 points on average.

### Synthesis of results

#### Primary and secondary outcomes: Conventional meta-analysis of direct comparisons

Table 2 displays meta-analyses with regards to primary and secondary outcomes at post-treatment. No specific type of treatment had a higher efficacy than other available comparison treatments. However, PCTs were less efficacious with regards to primary, but not secondary, outcomes than their available comparison treatments. Trauma focused treatments were in direct head-to-head comparisons to non-trauma focused treatments (TF vs. NTF) significantly more efficacious in primary outcomes. Heterogeneity appeared low for exposure therapies, for the head-to-head comparisons of TF vs. NTF and PCTs, but medium-to-high in both outcomes, and mostly significant according to the *Q* test, for CBT, prolonged exposure, and EMDR. Distinguishing between self-reports and clinician ratings did not reveal deviating results for any specific type of treatment (details omitted for brevity), but for the head-to-head comparisons of TF vs. NTF treatments: At post-treatment, TF treatments were more efficacious than NTF treatments in self-reports, *k* = 5, *g* = 0.21 [0.05, 0.37], *p* = .008, *Q* = 3.00, *p* = .557, *I*^2^ = 13 %.

**Table 2:** Meta-analyses of the efficacy of different types of treatment at post-treatment regarding primary and secondary outcomes

Treatments	*k*	Hedges’ *g*	*p*	*Q*	*p*	*I* ^2^
Cognitive behavior therapy (all)	15	−0.02 [−0.17, 0.14]	.843	21.89	.081	38 % (0–64 %)
		−0.03 [−0.18, 0.13]	.735	26.25	.024	50 % (5–70 %)
Trauma focused cognitive behavior therapy with exposure (subgroup)	10	0.03 [−0.15, 0.22]	.724	14.86	.095	42 % (42–88 %)
Exposure	10	0.03 [−0.17, 0.23]	.802	20.05	.018	59 % (59–90 %)
	7	0.17 [−0.04, 0.37]	.112	4.14	.658	0 % (0–58 %)
		−0.06 [−0.24, 0.13]	.556	6.22	.399	0 % (0–72 %)
Prolonged exposure	7	0.01 [−0.26, 0.28]	.927	14.98	.020	55 % (13–82 %)
		−0.02 [−0.39, 0.35]	.914	26.70	.0002	82 % (54–89 %)
Exposure + prolonged exposure	14	0.10 [−0.07, 0.26]	.240	19.33	.113	33 % (0–63 %)
		−0.02 [−0.20, 0.17]	.853	33.18	.002	57 % (31–78 %)
EMDR	6	0.01 [−0.45, 0.48]	.959	17.02	.005	70 % (35–87 %)
		0.17 [−0.32, 0.66]	.505	17.97	.003	73 % (39–87 %)
Present-centered therapies	6	−0.17 [−0.32, 0.00]	.038	4.38	.496	15 % (0–72 %)
		−0.04 [−0.15, 0.07]	.468	5.39	.370	0 % (0–77 %)
TF vs. NTF	9	0.14 [0.01, 0.27]	.030	7.31	.504	8 % (8–80 %)
		0.06 [−0.04, 0.15]	.269	9.16	.329	0 % (0–96 %)

*Note. k* = number of comparisons; *Q* = statistic of effect size heterogeneity. Values of Hedges’ *g* and *I* ^2^ are presented alongside their 95 % confidence intervals. Hedges’ *g* > 0 signifies a higher efficacy for the type of treatment of interest compared to all other available treatments, Hedges’ *g* < 0 signifies a lower efficacy. Per type of treatment, values in the first line pertain to primary outcomes (i.e., PTSD symptom severity), values in the second line to secondary outcomes (i.e., symptom severity of comorbid disorders, trauma-related symptoms, general symptom distress, social functioning, and quality of life)

Meta-analyses with regards to primary and secondary outcomes at follow-up (first and second follow-ups) are presented in Appendix (Table 8). TF treatments were again more efficacious than NTF treatments at both follow-ups. Pointing into the same direction, TFCBT + EX treatments appeared to be more efficacious with regards to primary outcomes at both follow-ups, but these results closely missed significance (*p*s = .053 and .076). PCTs were again less efficacious with regards to primary outcomes at first follow-up. Heterogeneity was again low for exposure therapies, TF vs. NTF, and PCTs, but medium-to-high for CBT, TFCBT + EX, prolonged exposure, and EMDR. Heterogeneity was also high for CBT at second follow-up. TF treatments were more efficacious than NTF treatments at first follow-up both in self-reports (*k* = 5, *g* = 0.15 [0.01, 0.29], *p* = .037, *Q* = 1.84, *p* = .766, *I*^2^ = 0 %) and in clinician ratings (*k* = 6, *g* = 0.18 [0.03, 0.33], *p* = .016, *Q* = 2.58, *p* = .765, *I*^2^ = 0 %).

#### Primary and secondary outcomes: the Wampold homogeneity test (WHT)

Primary and secondary outcomes at post-treatment were also investigated with the WHT. In 30 independent runs of random sign assignment with regards to primary outcomes, the *Q* test was only *twice* not significant (*p* > .05) and yielded in 28 replications a significant value (*p* < .05); the null hypothesis of no differences in treatment efficacy had to be rejected with *p* < .001 (binomial test). On average, *I*^2^ was 40 % across all replications, with a range of 28 % to 43 %. With regards to secondary outcomes, *Q* tests were significant in *all* of another 30 independent runs (*p* < .001); on average, *I*^2^ was 43 %, ranging from 35 % to 46 %. Restricting the study sample to studies that were also included by Benish et al. [23], we obtained 20 significant *Q* tests out of another 30 independent runs for primary outcomes (*p* < .001); on average, *I*^2^ was 38 %, ranging from 27 % to 43 %. In contrast, Benish et al. [23] reported a not significant *I*^2^ of 9.39 % for this sample of studies.

#### Clinical significance and dropout

Table 3 displays meta-analyses with regards to clinical significance and dropout at post-treatment. Additionally, the thresholds for clinically meaningful results [12] of *RR* ≤ 0.80 or *RR* ≥ 1.25 were considered for interpretation (see the Synthesis of results subsection in the Methods section): Where 95 % confidence intervals of risk ratios included these thresholds, results were interpreted as limited evidence of differential efficacy. Clinical significance could not be examined for exposure therapies and PCTs because of less than five comparisons available each.

**Table 3:** Meta-analyses of the efficacy of different types of treatment at post-treatment regarding clinical significance and dropout

Treatments	*k*	*RR*	*p*	*Q*	*p*	*I* ^2^
Cognitive behavior therapy (all)	11(7)	0.95 [0.83, 1.08]	.453	11.47	.322	0 % (0–47 %)
	15	1.20 [0.86, 1.68]	.281	24.17	.044	43 % (0–67 %)
Trauma focused cognitive behavior therapy with exposure (subgroup)	7	1.00 [0.85, 1.17]	.994	8.22	.223	0 % (0-94 %)
	10	1.26 [0.79, 2.01]	.341	18.73	.028	61 % (0-92 %)
Exposure	–	–	–	–	–	–
	7	1.12 [0.69, 1.79]	.666	3.01	.808	0 % (0–43 %)
Prolonged exposure	7(5)	1.13 [0.84, 1.52]	.415	16.05	.014	67 % (19–83 %)
	7	1.25 [0.76, 2.03]	.380	11.19	.083	39 % (0–75 %)
Exposure + prolonged exposure	9(6)	1.13 [0.89, 1.43]	.312	18.58	.017	58 % (13–79 %)
	14	1.22 [0.90, 1.67]	.201	14.91	.313	16 % (0–46 %)
EMDR	6(3)	1.06 [0.76, 1.48]	.733	14.51	.013	67 % (22–85 %)
	6	0.83 [0.53, 1.28]	.397	5.27	.384	0 % (0–77 %)
Present-centered therapies	–	–	–	–	–	–
	6	0.77 [0.33, 1.80]	.554	14.56	.012	82 % (22–85 %)
TF vs. non-TF	6(4)	1.31 [0.95, 1.79]	.095	9.69	.084	46 % (0–77 %)
	9	1.01 [0.53, 1.92]	.981	28.90	.0003	79 % (40–95 %)

*Note. k* = number of comparisons, with regards to clinical significance (first line), the number in parentheses refers to the number of comparisons that were based on recovery rates; *RR* = risk ratio; *Q* = statistic of effect size heterogeneity. *RR*s and *I* ^2^ are presented alongside their 95 % confidence intervals. *RR* > 1 signifies a higher rate of clinical significant change/dropout for the type of treatment of interest compared to all other available treatments, *RR* < 1 signifies a lower rate. Per type of treatment, values in the first line pertain to clinical significance (i.e., recovery rates or rates of patients improved), values in the second line to dropout

Treatments did not differ with regards to clinical significance or dropout. Trauma focused treatments appeared to have a higher efficacy than non-trauma focused treatments, but this result closely missed significance (*p* = .095). Heterogeneity was low for most treatments in clinical significance, but medium-to-high for prolonged exposure and EMDR. In contrast, heterogeneity was medium-to-high in dropout for most treatments, with the exception of EMDR, where heterogeneity was low.

With regards to the required five or more available comparisons, 13 studies reported rates of recovery or improvement at a first follow-up, while only four reported respective rates for two follow-ups [44, 59, 68, 71]. One overall follow-up was defined and investigated, using data from the second follow-up in studies that reported two follow-ups. PCTs could not be investigated at post-treatment; at follow-up PCTs appeared to be less efficacious, *k* = 5, *RR* = 0.79 [0.61, 1.02], *p* = .076, *Q* = 6.84, *p* = .144, *I*^2^ = 35 %, but this result closely missed significance. Trauma focused therapies appeared to be more efficacious at follow-up than non-trauma focued treatments, *k* = 6, *RR* = 1.21 [0.98, 1.49], *p* = .076, *Q* = 7.00, *p* = .221, *I*^2^ = 15 %, but this result also closely missed significance. The only other treatment type, for which five or more comparisons were available, was CBT. CBT did neither differ from other treatments concerning rates of recovery or improvement at follow-up, *k* = 9, *RR* = 1.12 [0.96, 1.30], *p* = .153, *Q* = 10.03, *p* = .263, *I*^2^ = 0 %; nor was the subgroup TFCBT + EX significantly more efficacious than comparison treatments, *k* = 7, *RR* = 1.14 [0.96, 1.34], *p* = .126, *Q* = 9.73, *p* = .136, *I*^2^ = 0 %.

#### Publication bias

Mostly, there was no indication that publication bias influenced results substantially (details omitted for brevity). However, with regards to primary outcomes, trim-and-fill added one missing study in comparisons of PCTs. This resulted in an overall treatment effect of *g* = −0.20 [−0.05, −0.35], *p* = .011, for PCTs which suggests that they were robustly less efficacious than other available comparison treatments. Trim-and-fill added two missing studies in comparisons of prolonged exposure with regards to clinical significance, three missing studies in comparisons of exposure therapies with regards to dropout, and one missing study in comparisons of prolonged exposure with regards to dropout. None of these corrections altered previous results substantially (details omitted for brevity).

#### Dependencies in the data

Excluding studies in cases where more than one comparison was reported [57] or where treatments of the same type were compared [46, 47, 58] did not change results substantially for any of the above reported analyses (details omitted for brevity). Thus, categorizing the hypnotherapy investigated in Brom et al. [57] as EX, which may appear debatable, did not influence overall results.

#### Sensitivity analyses

A number of studies were identified as influential cases in sensitivity analyses. Some influential studies were of higher quality and examined larger samples than the remaining studies [45, 59, 71], see Table 1. Hence, their exclusion may not be considered warranted in every comparison where they appeared to be influential. Others were of a comparably lower quality [62, 68], indicating that exclusion of these studies might be considered warranted in comparisons where they appeared influential.

With regards to primary outcomes at post-treatment, studies of Resick et al. [68] and Schnurr et al. [70, 71] appeared influential in the PE, EXPE and PCT categories. Excluding either the Resick et al. [68] or the Schnurr et al. [71] study rendered heterogeneity in comparisons of prolonged exposure not significant, *I*^2^ = 27 % and 29 %, respectively. Moreover, excluding the Resick et al. [68] study from comparisons of the combined EXPE category indicated a higher efficacy of treatments of this type, *g* = 0.19 [0.06, 0.33], *p* = .005, and diminished also heterogeneity to *I*^2^ = 2 %. Notably, the Resick et al. [68] study was of comparably low quality; hence, exclusion may be considered warranted, suggesting thus a true advantage of EXPE treatments over other treatments. Excluding either the Schnurr et al. [70] or the Schnurr et al. [71] study from comparisons of PCT rendered the overall treatment effect not significant, *g* = 0.16 [−0.10, 0.42], *p* = .220, and *g* = 0.09 [−0.08, 0.26], *p* = .290, respectively.

With regards to secondary outcomes at post-treatment, studies of Foa et al. [61], Power et al. [45], and, again, Resick et al. [68] were influential. Excluding either of these studies rendered heterogeneity in comparisons of CBT not significant, *I*^2^ = 46 %, 39 %, and 0 %, respectively.

With regards to clinical significance at post-treatment, studies of Ironson et al. [62], and, again, Schnurr et al. [70] and Schnurr et al. [71] were influential. Excluding the Schnurr et al. [71] study rendered heterogeneity in prolonged exposure and in the combined EXPE category not significant, *I*^2^ = 19 % and 14 %, respectively. Excluding the Ironson et al. [62] study rendered heterogeneity there also not significant, *I*^2^ = 39 % and 37 %, respectively, and resulted in limited evidence for a higher efficacy of these treatments, *RR* = 1.26 [1.02, 1.57], *p* = .035 (PE), and *RR* = 1.22 [1.01, 1.48], *p* = .043 (EXPE), respectively. As the Ironson et al. [62] study was of low quality, exclusion may be considered warranted, suggesting thus a true advantage of prolonged exposure over other treatments (the advantage of EXPE appeared to be driven by prolonged exposure). Furthermore, exclusion of the Schnurr et al. [70] study resulted in limited evidence for a higher efficacy of trauma focused treatments as well, *RR* = 1.46 [1.03, 2.08], *p* = .034. Finally, exclusion of the Cottraux et al. [59] study resulted in limited evidence for a higher risk of dropout for CBT, *RR* = 1.38 [1.11, 1.72], *p* = .005, and for TFCBT + EX, *RR* = 1.46 [1.14, 1.88], *p* = .003 and a lower risk of dropout for PCT, *RR* = 0.57 [0.43, 0.75], *p* < .001.

#### Moderator analyses

Moderator analyses revealed effects of study quality (Jadad rating) on primary outcomes at follow-up and of secondary outcomes at post-treatment in comparisons of CBT with other treatments. Studies with lower quality reported larger treatment differences, see Table 4. This pattern was also similar for secondary outcomes at post-treatment in comparisons of TFCBT + EX with other treatments (*QM* (3) = 13.59, *p* = .004; *QE*(7) = 7.20, *p* = .408; details omitted for brevity). Moreover, time since trauma was another moderator of secondary outcomes at post-treatment in comparisons of CBT with other treatments (slope = −0.09 [−0.16, −0.03], *p* = .003; *QM* (1) = 9.09, *p* = .003; *QE*(8) = 7.24, *p* = .512; similar for comparisons of TFCBT + EX with other treatments: slope = −0.15 [−0.23, −0.08], *p* < .001; *QM* (1) = 15.31, *p* < .001; *QE*(5) = 2.21, *p* = .820): Treatment differences were in favour of CBT (*g* > 0) in studies where time since trauma was less than four years, and in favour of comparative treatments (*g* < 0) where time since trauma was four or more than four years.

**Table 4:** Moderator analyses: efficacy of CBT according to study quality

	Primary outcomes at follow-up			Secondary outcomes at post-treatment		
Quality rating^a^	*k*	Hedges’ *g*	*p*	*k*	Hedges’ *g*	*p*
0	2	0.44 [0.77, 0.13]	.006	2	0.26 [−0.14, 0.66]	.196
1	3	−0.31 [−0.57, −0.04]	.023	3	−0.43 [−0.66, −0.18]	< .001
2	4	0.09 [−0.28,0,46]	.630	4	0.21 [−0.05, 0.46]	.108
3	1	−0.09 [−0.50, 0.33]	.672	1	0.03 [−0.38, 0.44]	.882
4	4	−0.09 [−0.25, 0.07]	.252	5	0.02 [−0.11, 0.15]	.744

*Note. k* = number of included studies. Values of Hedges’ *g* are presented alongside their 95 % confidence intervals. Hedges’ *g* > 0 signifies a higher efficacy for CBT compared to all other available treatments, Hedges’ *g* < 0 signifies a lower efficacy. Heterogeneity explained by quality rating in primary outcomes: *QM* (5) = 14.42, *p* = .013; residual heterogeneity: *QE*(9) = 10.88, *p* = .284; in secondary outcomes: *QM*(5) = 16.14, *p* < .001; *QE*(10) = 10.13, *p* = .429. ^a^ Quality rating according to Jadad et al. [39]

## Discussion

### Summary of evidence

In head-to-head comparisons between trauma focused treatments and non-trauma focused treatments, this meta-analysis revealed a significant difference in efficacy. Trauma focused treatments were slightly more efficacious with regards to PTSD symptom severity at post-treatment (*g* = 0.14), at the first (*g* = 0.17) and the second follow-up (*g* = 0.23). This result corresponds with results of other meta-analyses [12, 14], but this time based strictly on head-to-head comparisons of bona fide therapies only.

The results of this meta-analysis with regards to the specific types of therapy corroborate at a first glance that bona fide psychotherapies of PTSD appear mostly similar with regards to their efficacy [23]. There was no strong indication of cognitive behavioural therapy, exposure therapies, prolonged exposure, EMDR, or of trauma focused cognitive behavioural therapy to be more efficacious than any of the other available therapies. However, sensitivity analyses revealed that some of these null findings could be traced to low quality studies. Excluding these studies, we obtained evidence of an advantage of prolonged exposure and exposure therapies (*g* = 0.19) over other treatments considering PTSD symptom severity at post-treatment, and limited evidence of a higher efficacy of prolonged exposure (*RR* = 1.26) regarding recovery rates at post-treatment, cf. [16]. PCTs appeared somewhat less efficacious than other treatments with regards to PTSD symptom severity at post-treatment (*g* = −0.20; adjusting for publication bias) and at follow-up (*g* = −0.17), mirroring recent findings with regards to a somewhat diminished efficacy of bona fide supportive therapies also in the treatment of depression [27]. However, PCTs were equally effective with regards to secondary outcomes as their available comparison treatments. No significant and clinically meaningful differences with regards to dropout were observed. All differential treatment effects were at most only small in size [53] and far below proposed thresholds for clinically meaningful differences [12, 54].

Application of the omnibus test of overall effect size heterogeneity (see Benish et al. [23]) indicated that the null hypothesis of no treatment differences had to be rejected in the full study sample; effect size heterogeneity was larger than expected by chance. Similarly, this null hypothesis had to be rejected in the study sample of Benish et al. [23]. Our analysis was based on an improved application of this analytic method, using a Monte Carlo permutation approach and basing our conclusions on 30 independent runs of the Wampold homogeneity test, and differed also by utilizing a stricter definition regarding primary outcomes. Despite these differences, the obtained results cast doubt on the results of the Benish et al. [23] meta-analysis, but also on other meta-analyses that used this method [4, 24, 25, 33]. We thus caution on the inconsiderate application of the omnibus test of overall effect size heterogeneity and recommend a Monte Carlo permutation approach, as used in the present study, also in future applications.

Substantial effect size heterogeneity was also apparent in conventional meta-analyses of specific treatment types and sensitivity analyses revealed that it could be traced to a number of influential studies. The Schnurr et al. [71] study was identified as influential, being subject to controversy before [29, 30], regarding the bona fide status of the PCT investigated in one arm of this study. Yet, we also identified a number of other, apparently less controversial, studies. Some of these [45, 59, 70] had a higher study quality and often larger patient samples than remaining studies, indicating that their exclusion may not be considered warranted. Studies of Ironson et al. [62] and Resick et al. [68] were of comparably lower quality, suggesting that their exclusion may be considered warranted.

Excluding influential studies, exposure therapies and prolonged exposure appeared more efficacious than other treatments with regards to PTSD symptom severity and recovery rates, whereas PCTs appeared equally efficacious as other treatments with regards to PTSD symptom severity. As the latter finding depended on the exclusion of large and high-quality studies [70, 71], we deem them likely biased. Moreover, systematic effects of study quality were also observed in comparisons involving cognitive behavioural therapy; differences at post-treatment and follow-up were larger in studies with lower quality. Lastly, we obtained evidence of publication bias, that, when adjusted for, revealed as its most significant result that PCTs were robustly less efficacious than other treatments with regards to PTSD symptom severity.

Study quality appeared thus influential with regards to aggregated tests of differential efficacy of bona fide psychotherapies of PTSD. However, based on the available evidence, obtained results appeared fickle and need to be interpreted with caution. Even though another seven studies over-and-above the 15 studies investigated by Benish et al. [23] were identified and included in the present meta-analysis, as much as nine (41 %) of all included studies examined less than 20 patients within one or both treatment arms, and another five (23 %) less than 30 patients; study quality appeared, overall, only mediocre. In order to be able to draw firmer conclusions, more high-quality studies using ITT analyses are still needed in this field of research.

As the current debate on the relative efficacy of PTSD treatments focused a lot on the topic of whether individual studies should be included or excluded in meta-analysis [29, 30], the sensitivity analyses reported here show that this factor could actually be decisive in terms of the overall meta-analytical result regarding equal or different efficacy of compared treatments. Concerning the consequences with regards to the dodo bird verdict and the question of what works in psychotherapy, we thus recommend a refinement and clarification of the bona fide definition which should ideally result in a clear decision rule with regards to the inclusion and exclusion of especially supportive and present-centered therapies in analysis. Otherwise, the selection of study samples may overly depend on the theoretical position of the study authors, or meta-analyses from authors with different theoretical positions might not be adequately comparable.

### Limitations

Using additional terms in the literature search, like the names of specific psychotherapies (e.g., “CBT”, “exposure therapy” or “EMDR”), might have provided additional studies that were overlooked using the present search terms.

Treatment categorizations adhered to the descriptions provided in the studies themselves, which may have introduced spurious heterogeneity with regards to categorization and may have impacted on our results. Moreover, there were too few studies for most of the specified treatment types to systematically test effect moderation with moderator analysis. Thus, effect size heterogeneity that could not be traced to influential studies could mostly not be examined with regards to proposed moderator variables. This affected specifically meta-analyses on EMDR, where heterogeneity was substantial and could not be traced to single influential studies.

The assessment of eligibiliy criteria, in particular the bona fide status of treatments, is still a major point of discussion. Hence, independent codings of a second rater, in addition to comparing codings of the present study with codings provided in other meta-analyses, might have benefitted the reliability of our decisions to include or exclude studies in the present meta-analysis.

Studies were very variable with regards to time of follow-up. Bradley et al. [13] suggested limiting comparisons at follow-up to assessments that took place six or more months following end of treatment. In the present study, all available follow-ups were used for analysis, regardless of exact time of follow-up, which may have influenced results. Specifically with regards to longer follow-up periods results could be confounded, because patients might have sought additional, external, treatment. This needs to be controlled both in primary studies and meta-analytical investigations. However, not enough information was available in primary studies to investigate this topic in the present meta-analysis.

Distinguishing different types of recovery and improvement might accentuate differences in efficacy, particularly because improvement is defined by study authors in different ways [13, 74]. As only nine studies reported recovery rates at post-treatment, combining recovery and improvement rates allowed the assessment of clinical significance, but may have influenced results.

Effects of researcher allegiance were neither examined nor controlled for in the present study. Researcher allegiance may affect differences in treatment efficacy and was reported to explain 12 % of variation in outcome regarding various trauma focused treatments [33]. However, allegiance failed to explain differences of efficacy in a number of other meta-analyses [22, 27, 75], and it is not clear whether allegiance ratings reliably reflect true differences in allegiance [76] or how well meta-analytical reviews may adjust for it.

Considering public health issues, a variety of topics not considered here may still have to be evaluated with regards to the comparative efficacy of treatments [48]. Cost of treatments, the level of education and the intensity of training of therapists, the preferences of patients and the applicability of treatments for a wide range of patients may influence what works best for patients as well as what is affordable to implement in public health care. For example, present-centered therapies could be easier to deliver at a lower cost to more varied patients with a less skilled workforce, and these advantages might compensate for a lower efficacy with regards to health outcome. More research is needed here.

More research on traumatized populations with severe comorbidities, like psychosis (mostly an exclusion criteria in PTSD studies), as well as on patients with additional and multiple life burdens and comorbidities, such as homelessness, unemployment, suicidality or with a criminal justice involvement, is also needed [48]. Information on the efficacy of PTSD treatments in such highly vulnerable populations is currently lacking.

## Conclusions

The bona fide distinction is an interesting and promising research heuristic that is increasingly and independently used in a number of meta-analyses [22, 27, 28], even though its definition and criteria may need to be extended or specified more clearly [10, 30]. The stated goal of the present study was to apply this distinction, but not to improve it. On these premises, evidence for at most small differences in efficacy between bona fide psychotherapies for PTSD was obtained. Differences were far below proposed thresholds of clinically meaningful differences, but corroborate that trauma focused treatments and prolonged exposure and exposure therapies are slightly more efficacious than other therapies. While the lower efficacy of present-centered therapies mirrors recent findings in the field of treatment of depression, more high-quality research is still needed to draw firm conclusions. Factors that may affect and that may broaden the scope of treatment efficacy, such as public health issues and the requirements of highly vulnerable populations, are understudied in available research and need to be considered in future research.

## Appendix

Additionial Data and Results.

**Table 5:** Primary outcome measures

		# Studies	Used in studies
Interviews			
CAPS	Clinician Administered PTSD Scale (Blake et al., 1995)	14	44–47, 58, 64, 66–73
PSS-I	PTSD Symptom Scale (Foa, Riggs, Dancu, & Rothbaum, 1993)	2	42, 61
CGI	Clinical Global Impression of PTSD (Guy, 1976)	1	46
PTSD-I	PTSD Interview (Watson, Juba, Manifold, Kucala, & Anderson, 1991)	1	60
SI-PTSD	Davidson’s Structured Interview for PTSD (Davidson, Smith, & Kudler, 1989)	1	63
Self-report scales			
IES/IES-R	Impact of Events Scale (Horowitz, Wilner, & Alvarez, 1979)	9	45, 46, 57, 58, 60, 63, 66, 69, 72
PSS-SR	PTSD Symptom Scale Self-Report (Foa et al., 1993)	5	60, 62, 66, 68, 69
PCL	PTSD Checklist (Weathers, Litz, Herman, Huska, & Keane, 1993)	4	47, 59, 70, 71
CMS	Civilian Mississippi Scale for PTSD (Keane, Caddell, & Taylor, 1988)	1	60
MMPI-K	Keane’s Post-Traumatic Stress Disorder Scale from the Minnesota Multiphasic Personality Inventory (Keane, Malloy, & Fairbank, 1984)	1	63
PENN	Penn Inventory (Hammarberg, 1992)	1	72
PDS	Post-traumatic Stress Diagnostic Scale (Foa, 1995)	1	65
SI-PTSD	Davidson’s Structured Interview for PTSD, self-report version (Davidson et al., 1989)	1	45

**Table 6:** Secondary outcome measures

		# Studies	Used in studies
Measures of depression, anxiety, substance abuse, and of trauma-related guilt, anger, dissociation, and cognitions			
BDI	Beck Depression Inventory (Beck, Ward, Mendelsohn, Mock, & Erbaugh, 1961)	16	42, 44, 47, 58–63, 66–69, 71–73
HADS	The Hospital Anxiety and Depression Scale (Zigmond & Snaith, 1983)	1	45
HAM-D	Hamilton Rating Scale for Depression (Hamilton, 1960)	1	66
MADRS	Montgomery Asberg Depression Rating Scale (Montgomery & Asberg, 1979)	1	45
SRQ-20	Self-Reporting Questionnaire 20 (Harding et al., 1980)	1	65
STAI	State-Trait Anxiety Inventory (Spielberger, Gorsuch, & Lushene, 1970)	9	42, 44, 57, 58, 60, 61, 66, 69, 71
HAM-A	Hamilton Rating Scale for Anxiety (Hamilton, 1959)	3	45, 59, 66
BAI	Beck Anxiety Inventory (Beck, Epstein, Brown, & Steer, 1988)	2	66, 72
FQ	Fear Questionnaire (Marks & Mathews, 1979)	1	59
ASI	Addiction Severity Index (McLellan et al., 1992)	1	71
SUI	Substance Use Inventory (Sobell et al., 1980)	1	46
RAST	Rape Aftermath Symptom Test (Kilpatrick, 1988)	1	42
NFQ	Night Frequency Questionnaire (Krakow et al., 2000)	1	47
PSQI	Pittsburgh Sleep Quality Index (Germain, Hall, Krakow, Shear, & Buysse, 2005)	1	47
NES	Nightmare Effects Survey (Krakow et al., 1996)	1	47
TRGI	Trauma-Related Guilt Inventory (Kubany et al., 1996)	1	68
COOK	Cook-Medley Hostility Scale (Cook & Medley, 1954)	1	44
STAXI	State-Trait Anger Expression Inventory (Spielberger, 1988)	2	44, 57
DES(−II)	Dissociative Experiences Scale (Bernstein & Putnam, 1986)	2	44, 69
CCQ	Catastrophic Cognitions Questionnaire (Khawaja & Oei, 1992)	1	58
WAS	World Assumptions Scale (Janoff-Bulman, 1989)	1	66
Measures of quality of life, general symptom distress, and social functioning			
QOLI	Quality of Life Inventory (Frisch, Cornell, Villanueva, & Retzlaff, 1992)	3	44, 66, 71
QOLS	Quality of Life Scale (Marks et al., 1993)	1	59
GHQ 28	General Health Questionnaire (Goldberg & Hillier, 1979)	2	70, 72
SF-36/12	Short-Form Health Survey (Ware & Sherbourne, 1992)	4	47, 65, 70, 71
SCL-90 (R)	Symptom Checklist-90, Dutch version (Arindell & Ettema, 1981)	2	57, 60
SHEEHAN	The Sheehan Disability Scale (Sheehan, 1986)	1	45
SAS	Social Adjustment Scale (Weissman & Paykel, 1974)	1	61

*Note*. Listed are references to the original instruments, which may differ from references provided in the studies themselves

**Table 7:** Treatment categorizations of included studies in the present meta-analysis compared to previous meta-analyses

		Meta-analysis						
Study	Treatments	Present	Powers et al. (2010)	Bisson et al. (2007)	Seidler & Wagner (2006)	Bradley et al. (2005)	Davidson & Parker (2001)	Commentary
Brom et al. [57]	Trauma desensitization	EX (TF)	–	TFCBT^a^	–	EX	–	Brom et al. [57], p. 607: “The emphasis of the hypnotherapists in our study was on behavioral therapy. The goal was to bring the patient in contact with the reality of the traumatic event and to bring about a decrease in the conditioned responses triggered by the event.”
	Brief psychodynamic therapy	No specific category	–	Other^b^	–	Other	–	
	Hypnotherapy	EX (TF)	–	Other^b^	–	Other	–	
Devilly & Spence [60]	Cognitive-behavior trauma treatment protocol	CBT (TF) (TFCBT + EX)	–	TFCBT^a^	TFCBT	CBT + EX	In vivo exposure or CBT	
	EMDR	EMDR (TF)	–	EMDR^a^	EMDR	EMDR	EMDR	
Foa et al. [42]	Stress inoculation training	CBT	Active treatment	Stress manage-ment^b^	–	CBT	–	Inoculation training (Meichenbaum, 1974) includes education, muscle relaxation training, breathing retraining, role playing, covert modeling, guided self-dialogue, thought stopping (see Rothbaum et al., 2000). No instructions for exposure were included, see [42].
	Prolonged exposure	PE (TF)	PE	TFCBT^a^	–	EX	–	
	Supportive counseling	Non-bona fide (excluded)	Psych. placebo	Other therapies^b^	–	–	–	
Foa et al. [61]	Stress inoculation training (SIT)	CBT	Active treatment	Stress manage-ment (SM) ^b^	–	CBT	–	
	Prolonged exposure	PE (TF)	PE	TFCBT^a^	–	EX	–	
	Prolonged exposure + stress inoculation training	Component treatment (excluded)	PE + SIT	TFCBT^a^ + SM	–	–	–	
Ironson et al. [62]	EMDR	EMDR (TF)	–	EMDR^a^	EMDR	EMDR	–	
	Prolonged exposure	PE (TF)	–	TFCBT^a^	TFCBT	EX	–	
Lee et al. [63]	Stress inoculation training + prolonged exposure	CBT (TF) (TFCBT + EX)	–	Stress manage-ment + TFCBT^a^	TFCBT	CBT + EX	–	
	EMDR	EMDR (TF)	–	EMDR^a^	EMDR	EMDR	–	
Marks et al. [64]	Cognitive restructuring	CBT (TF)	Active treatment	TFCBT^a^	–	CBT	–	
	Prolonged exposure	PE (TF)	PE	TFCBT^a^	–	EX	–	
	Cognitive restructuring + prolonged exposure	Component treatment (excluded)	PE + CR	TFCBT^a^	–	–	–	
	Relaxation	Non-bona fide (excluded)	Psych. placebo	Stress manage-ment^b^	–	–	–	
McDonagh et al. [44]	CBT	CBT (TF) (TFCBT + EX)	PE	–	–	–	–	McDonagh et al. [44] investigated a combination of exposure therapy and cognitive restructuring and consider their treatment as CBT and as an extension of PE. Cognitive elements are not considered elements of prolonged exposure, see [42].
	Problem-solving therapy	PCT	Active treatment	–	–	–	–	
Paunovic & Öst [66]	CBT	CBT (TF) (TFCBT + EX)	–	TFCBT^a^	–	CBT + EX	–	
	Exposure	EX (TF)	–	TFCBT^a^	–	EX	–	
Power et al. [45]	Cognitive restructuring + exposure	CBT (TF) (TFCBT + EX)	PE + CR	TFCBT^a^	TFCBT	–	–	
	EMDR	EMDR (TF)	Active treatment	EMDR^a^	EMDR	–	–	
Resick et al. [68]	Cognitive processing	CBT (TF) (TFCBT + EX)	Active treatment	TFCBT^a^	–	CBT + EX	–	
	Prolonged exposure	PE (TF)	PE	TFCBT^a^	–	EX	–	
Rothbaum et al. [69]	EMDR	EMDR (TF)	Active treatment	EMDR^a^	EMDR	–	–	
	Prolonged exposure	PE (TF)	PE	TFCBT^a^	TFCBT	–	–	
Schnurr et al. [70]	Trauma-focused group therapy	CBT (TF) (TFCBT + EX)	–	Group TFCBT^a^	–	CBT + EX		
	Present-centered group therapy	PCT	–	Group CBT^b^	–	Supportive control condition		
Schnurr et al. [71]	Present-centered therapy	PCT	Psych. placebo	–	–	–	–	
	Prolonged exposure	PE (TF)	PE	–	–	–	–	
Tarrier et al. [72]	Cognitive therapy	CBT (TF)	–	TFCBT^a^	–	CBT	–	
	Imaginal exposure	EX (TF)	–	TFCBT^a^	–	EX	–	
Taylor et al. [73]	EMDR	EMDR (TF)	Active treatment	EMDR^a^	EMDR	EMDR	–	Taylor et al. [73] call one of their treatments exposure therapy; this treatment differs from prolonged exposure, see [42]. Half of the sessions were used for in vivo exposition under supervision of the therapists.
	Exposure therapy	EX (TF)	PE	TFCBT^a^	TFCBT	EX	–	
	Relaxation training	Non-bona fide (excluded)	Psych. placebo	Stress manage-ment^b^	–		–	

*Note*. Wait-list controls are not listed in this table. Bisson et al. [11], p. 103: ^a^“Treatments delivered on an individual basis that focused on the memory for the traumatic event and its meaning.”/ ^b^“Treatments delivered on an individual basis that do not place the main focus of treatment on the trauma.”

**Table 8:** Meta-analyses of the efficacy of different types of treatment at follow-up regarding primary and secondary outcomes

Treatments	*k*	Hedges’ *g*	*p*	*Q*	*p*	*I* ^2^
First follow-up						
Cognitive behavior therapy (all)	14	0.01 [−0.17, 0.20]	.906	24.64	.026	49 % (5–71 %)
		−0.02 [−0.16, 0.12]	.739	20.95	.074	28 % (0–66 %)
Trauma focused cognitive behavior therapy with exposure (subgroup)	9	0.13 [0.00, 0.26]	.053	14.88	.062	0 % (0–93 %)
		0.07 [−0.12, 0.25]	.483	14.67	.066	40 % (40–93 %)
Exposure	7	−0.04 [−0.25, 0.17]	.705	2.88	.824	0 % (0–40 %)
		−0.06 [−0.25, 0.13]	.506	5.96	.428	0 % (0–71 %)
Prolonged exposure	7	0.10 [−0.14, 0.34]	.406	9.43	.151	39 % (0–70 %)
		0.18 [−0.12, 0.47]	.240	14.06	.029	62 % (7–80 %)
Exposure + prolonged exposure	14	0.04 [−0.11, 0.20]	.575	13.67	.398	20 % (0–57 %)
		0.06 [−0.11, 0.22]	.515	21.89	.057	39 % (0–67 %)
EMDR	5	−0.02 [−0.62, 0.57]	.940	13.32	.010	72 % (28–87 %)
		−0.05 [−0.66, 0.56]	.874	13.65	.009	73 % (17–84 %)
Present-centered therapies	6	−0.17 [−0.30, −0.04]	.010	3.71	.592	0 % (0–67 %)
		−0.05 [−0.18, 0.09]	.477	7.26	.202	13 % (0–67 %)
TF vs. NTF	9	0.17 [0.05, 0.28]	.006	4.05	.852	0 % (0–67 %)
		0.05 [−0.07, 0.18]	.401	11.70	.165	18 % (18–94 %)
Second follow-up						
Cognitive behavior therapy (all)	8	0.02 [−0.40, 0.44]	.926	25.14	.001	80 % (44–86 %)
		−0.06 [−0.37, 0.24]	.680	23.12	.002	72 % (39–85 %)
Trauma focused cognitive behavior therapy with exposure (subgroup)	5	0.30 [−0.03, 0.63]	.076	7.95	.093	52 % (52–96 %)
		0.16 [−0.11, 0.43]	.234	8.69	.070	52 % (52–94 %)
TF vs. NTF	6	0.23 [0.01, 0.46]	.045	8.20	.146	28 % (28–95 %)
		0.05 [−0.08, 0.18]	.466	5.35	.374	0 % (0–92 %)

*Note. k* = number of comparisons; *Q* = statistic of effect size heterogeneity. Values of Hedges’ *g* and *I* ^2^ are presented alongside their 95 % confidence intervals. Hedges’ *g* > 0 signifies a higher efficacy for the type of treatment of interest compared to all other available treatments, Hedges’ *g* < 0 signifies a lower efficacy. Per type of treatment, values in the first line pertain to primary outcomes (i.e., PTSD symptom severity), values in the second line to secondary outcomes (i.e., symptom severity of comorbid disorders, trauma-related symptoms, general symptom distress, social functioning, and quality of life)

## Additional file

Additional file 1: Studies that were excluded from Meta-Analysis. (DOCX 20 kb)

## Footnotes

1 (a) Was the study described as randomized? (b) Was the study described as double blind? (c) Was there a description of withdrawals and dropouts? For further information on the appropriate fulfillment of each criteria and scoring, see [39].

2 The study of Blanchard et al. [1] is of special interest concerning the validity of the bona fide criteria for the identification of active treatments. The authors describe both treatments as active treatments giving reasonable descriptions. Both treatments proved effective, but one of them did not mention the necessary two of the four operational bona fide criteria (citation of approach, reference to psychological process, treatment manual, active ingredients with citation). This study is therefore a good example as to why it may be necessary to develop more valid operational criteria (see Discussion).

3 If a study presented comparisons between different active treatments and between treatment components, only the comparisons between the different active treatments were included (see Table 1); comparisons between treatment components were excluded. The mentioned excluded studies contained only comparisons between treatment components.
