# Supplementary material for: The relative efficacy of bona fide psychotherapies for post-traumatic stress disorder: a meta-analytical evaluation of randomized controlled trials
Source: BMC Psychiatry. 2016 Jul 26;16:266. doi: 10.1186/s12888-016-0979-2 (PMC4962479; doi:10.1186/s12888-016-0979-2)
Supplement: Additional file 1: — Studies that were excluded from Meta-Analysis. (DOCX 20 kb) [file 12888_2016_979_MOESM1_ESM.docx]

**Appendix B**

Studies that were excluded from meta-analysis, because they compared treatments that were not bona fide:

[1] Blanchard, E. B., Hickling, E. J., Devineni, T., Veazey, C. H., Galovski, T. E., Mundy, E., … Buckley, T. C. (2003). A controlled evaluation of cognitive behavioral therapy for posttraumatic stress in motor vehicle accident survivors. *Behaviour Research and Therapy, 41,* 79-96. doi: 10.1016/S0005-7967(01)00131-0

[2] Brett, L., Engel, C. S., Bryant, R. A., & Papa, A. (2007). A randomized controlled proof-of-concept trial of an Internet-based, therapist-assisted self-management treatment for posttraumatic stress disorder. *The American Journal of Psychiatry, 164*, 1676-1683. doi: 10.1176/ appi.ajp.2007.06122057

[3] Hien, D. A., Wells, E. A., Jiang, H., Suarez-Morales, L., Campbell, A. N. C., Cohen, L. R., … Nunes, E. V. (2009). Multisite randomized trial of behavioral interventions for women with co-occuring PTSD and substance use disorders. *Journal of Consulting and Clinical Psychology, 77*, 607-619. doi: 10.1037/a0016227

[4] Holzapfel, S., Blanchard, E. B., Hickling, E. J., & Malta, L. S. (2005). A crossover evaluation of supportive psychotherapy and cognitive behavioral therapy for chronic PTSD in motor vehicle accident survivors. In M. E. Abelian (Ed.), *Focus on psychotherapy research* (pp. 207-218). Hauppauge, NY: Nova Science Publishers.

[5] Neuner, F., Onyut, P. L., Ertl, V., Odenwald, M., Schauer, E., & Elbert, T. (2008). Treatment of posttraumatic stress disorder by trained lay counselors in an African refugee settlement: A randomized controlled trial. *Journal of Consulting and Clincal Psychology, 76*, 686-694. doi: 10.1037/0022-006X.76.4.686

Studies that were excluded from meta-analysis, because they were component or dismantling studies:

[6] Beidel, D. C., Frueh, B. C., Uhde, T. W., Wong, N., & Mentrikoski, J. M. (2011). Multicomponent behavioral treatment for chronic combat-related posttraumatic stress disorder: A randomized controlled trial. *Journal of Anxiety Disorders, 25*, 224-231. [doi:10.1016/j.janxdis.2010.09.006](http://dx.doi.org/10.1016/j.janxdis.2010.09.006)

[7] Bryant, R. A., Moulds, M. L., Guthrie, R. M., Dang, S. T., & Nixon, R. D. V. (2003). Imaginal exposure alone and imaginal exposure with cognitive restructuring in treatment of posttraumatic stress disorder. *Journal of Consulting and Clinical Psychology, 71,* 706-712. doi: [10.1037/0022-006X.71.4.706](http://psycnet.apa.org/doi/10.1037/0022-006X.71.4.706)

[8] Cloitre, M., Stovall-McClough, K. C., Nooner, K., Zorbas, P., Cherry, S., Jackson, C. L., … Petkova, E. (2010). Treatment for PTSD related to childhood abuse: A randomized controlled trial. *The American Journal of Psychiatry, 167*, 915-924. doi: 10.1176/ appi.ajp.2010.09081247

[9] Foa, E. B., Hembree, A., Cahill, S. P., Rauch, S. A. M., Riggs, D. S., Feeny, N. C., & Yadin, E. (2005). Randomized trial of prolonged exposure for posttraumatic stress disorder with and without cognitive restructuring: Outcome at academic and community clinics. *Journal of Consulting and Clinical Psychology, 73,* 953-964. doi: 10.1037/0022-006X.73.5.953

[10] Glynn, S. M., Eth, S., Randolph, E. T., Foy, D. W., Urbaitis, M., Boxer, L., … Corthers, J. (1999). A test of behavioral family therapy to augment exposure for combat-related posttraumatic stress disorder. *Journal of Consulting and Clinical Psychology, 67,* 243-251. doi: [10.1037/0022-006X.67.2.243](http://psycnet.apa.org/doi/10.1037/0022-006X.67.2.243)

[11] Resick, P. A., Galovski, T. E., O’Brien Uhlmansiek, M., Scher, C. D., Clum, G. A., & Young-Xu, Y. (2008). A randomized clinical trial to dismantle components of cognitive processing therapy for posttraumatic stress disorder in female victims of interpersonal violence. *Journal of Consulting and Clinical Psychology, 76,* 243-258. doi: 10.1037/0022-006X.76.2.243

[12] Roy, M. J., Law, W., Patt, I., Difede, J., Rizzo, A., Graap, K., & Rothbaum, B. (2006). Randomized controlled trial of CBT with virtual realitiy exposure therapy for PTSD. *Annual Review of CyberTherapy and Telemedicine, 4*, 39-44.

Studies that were excluded from meta-analysis, because they reported preliminary analyses of studies that were not included in the present meta-analysis themselves:

[13] Classen, C., Koopman, C., Nevill-Manning, K., & Spiegel, D. (2001). A preliminary report comparing trauma-focused and present-focused group therapy against a wait-listed condition among childhood sexual abuse survivors with PTSD. *Journal of Aggression, Maltreatment & Trauma, 4,* 265-288. doi: 10.1300/J146v04n02_12

Studies that were excluded from meta-analysis, because they reported additional analyses on data of studies already included in the present meta-analysis:

[14] Ginzburg, K., Butler, L. D., Giese-Davis, J., Cavanaugh, C. E., Neri, E., Koopman, C., … Spiegel, D. (2009). Shame, guilt, and posttraumatic stress disorder in adult survivors of childhood sexual abuse at risk for human immunodeficiency virus. *The Journal of Nervous and Mental Disease, 197*, 536-542. doi: 10.1097/ NMD.0b013e3181ab2ebd

[15] Nishith, P., Nixon, R. D. V., & Resick, P. A. (2005). Resolution of trauma-related guilt following treatment of PTSD in female rape victims: A result of cognitive processing therapy targeting comorbid depression? *Journal of Affective Disorders, 86*, 259-265. doi: 10.1016/j.jad.2005.02.013

[16] Stapleton, J. A., Taylor, S., & Asmundson, G. J. G. (2006). Effects of three PTSD treatments on anger and guilt: Exposure Therapy, Eye Movement Desensitization and Reprocessing, and Relaxation Training. *Journal of Traumatic Stress, 19*, 19-28. doi: 10.1002/jts.20095

[17] Thrasher, S., Power, M., Morant, N., Marks, I., & Dalgleish, T. (2010). Social support moderates outcome in a randomized controlled trial of Exposure Therapy and (or) Cognitive Restructuring for chronic posttraumatic stress disorder. *The Canadian Journal of Psychiatry, 55*, 187-265.

Studies that were excluded from meta-analysis, because they did not examine adults and/or did not ascertain a PTSD diagnosis according to DSM-III or DSM-IV:

[18] Classen, C. C., Palesh, O. G., Cavanaugh, C. E., Koopman, C., Kaupp, J. W., Kraemer, H. C., … Spiegel, D. (2011). A comparison of trauma-focused and present-focused group therapy for survivors of childhood sexual abuse: A randomized controlled trial. *Psychological Trauma: Research, Practice, and Policy, 3*, 84-93. doi: 10.1037/a0020096

[19] Schaal, S., Elbert, T., & Neuner, F. (2009). Narrative Exposure Therapy versus Interpersonal Psychotherapy. *Psychotherapy and Psychosomatics, 78*, 298-306. doi: 10.1159/000229768

[20] Scheck, M. M., Schaeffer, J. A., & Gillette, C. (1998). Brief psychological intervention with traumatized young women: the efficacy of eye movement desensitization and reprocessing. *Journal of Traumatic Stress, 11,* 25-44.

Studies that were excluded from meta-analysis, because they included treatments that were delivered in a standard protocol that could not be adapted to individual patients:

[21] Carlson, J. G., Chemtob, C. M., Rusnak, K., Hedlund, N. L., & Muraoka, M. Y. (1998). Eye movement desensitization and reprocessing (EMDR) treatment for combat-related posttraumatic stress disorder. *Journal of Traumatic Stress, 11,* 3-24.

[22] Echeburúa, E., de Corral, P., Zubizarreta, I., & Sarasua, B. (1997). Psychological treatment of chronic posttraumatic stress disorder in victims of sexual aggression. *Behavior Modification, 21,* 433-456.

[23] Vaughan, K., Armstrong, M. S., Gold, R., O’Connor, N., Jenneke, W., & Tarrier, N. (1994). A trial of eye movement desensitization compared to image habituation training and applied muscle relaxation in post-traumatic stress disorder. *Journal of Behavior Therapy & Experimental Psychiatry, 25,* 283-291.doi: 10.1016/0005-7916(94)90036-1

Studies that were excluded from meta-analysis, because they examined only two participants in one treatment arm:

[24] Gamito, P., Oliveira, J., Rosa, P., Morais, D., Duarte, N., Oliveira, S., & Saraiva, T. (2010). PTSD elderly war veterans: A clinical controlled pilot study. *Cyberpsychology, Behavior, and Social Networking, 13*, 43-48. doi: 10.1089/cyber.2009.0237
